# Psilocybin restores behavior and 5-HT_2A_ signaling while reducing microglial density after chronic traumatic brain injury in rats

**DOI:** 10.1016/j.xcrm.2026.102867

**Published:** 2026-06-12

**Authors:** Josh Allen, Bianca Jupp, Tamara L. Baker, Mohammad B. Haskali, Robert Brkljača, Zoe Plummer, Mujun Sun, Justin Brand, Brian R. Christie, Chantel T. Debert, Stuart J. McDonald, Terence J. O’Brien, Pablo M. Casillas-Espinosa, Sandy R. Shultz

**Affiliations:** 1 Department of Neuroscience, School of Translational Medicine, Monash University, Melbourne, VIC, Australia; 2 Centre for Trauma and Mental Health Research, Vancouver Island University, Nanaimo, BC, Canada; 3 School of Medical Sciences, Faculty of Health, University of Victoria, Victoria, BC, Canada; 4 Sir Peter MacCallum Department of Oncology, University of Melbourne, Melbourne, VIC, Australia; 5 Department of Radiopharmaceutical Sciences, Cancer Imaging, The Peter MacCallum Cancer Centre, Melbourne, VIC, Australia; 6 Department of Clinical Neuroscience, Cumming School of Medicine, University of Calgary, Calgary, AB, Canada; 7 Institute on Aging & Lifelong Health, University of Victoria, Victoria, BC, Canada

**Keywords:** concussion, fluid-percussion injury, psychedelic, positron emission tomography, PET, serotonin receptor, microglia, IBA1

## Abstract

Traumatic brain injury (TBI) causes persistent neurobehavioral deficits and increases the risk of psychiatric disorders, including depression, anxiety, and cognitive dysfunction linked to disrupted neuroplasticity, neuroinflammation, and serotonergic (5-HT) signaling. No effective pharmacotherapies exist for chronic TBI. Psilocybin, a psychedelic 5-HT_2A_ receptor agonist, shows promise due to its neuroplasticity-enhancing, anti-inflammatory, and antidepressant effects. Here, male rats received fluid-percussion or sham injury, followed one year later by a single psilocybin (1 mg/kg) or saline injection. Behavioral testing began 24 h later, and positron emission tomography assessed 5-HT_2A_ binding after two weeks. TBI produced persistent sensorimotor, learning and memory, and affective deficits; reduced 5-HT_2A_ binding; and microglial alterations in the medial prefrontal cortex characterized by decreased process branching and enlarged soma size. Psilocybin treatment could improve sensorimotor function, restore 5-HT_2A_ binding, and reduce microglial cell counts. These findings highlight psilocybin’s therapeutic potential in chronic TBI and support further investigation of psychedelic treatments.

## Highlights

• Psilocybin restores TBI-induced behavioral deficits
• Deficits in 5-HT_2A_ receptor binding induced by TBI are reversed by psilocybin
• Psilocybin decreases the number of microglial cells in the prefrontal cortex

## Graphical abstract

**Figure:**
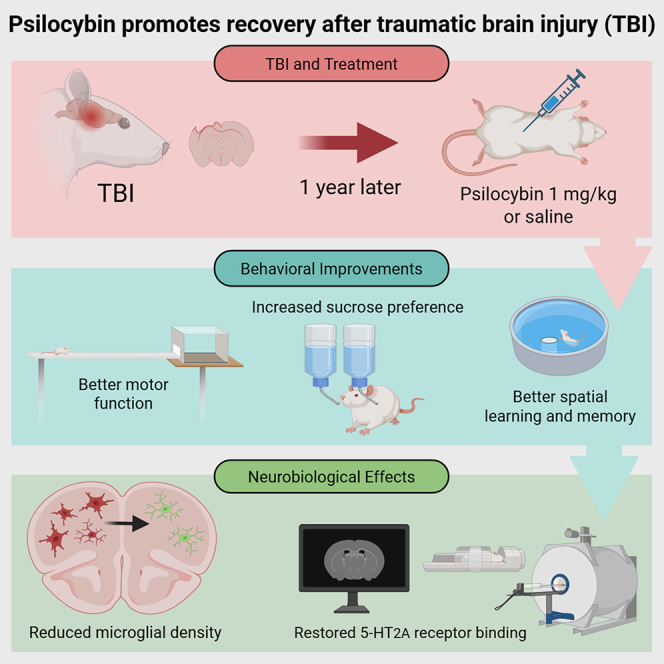

## Teaser

Allen et al. show that a single psilocybin injection administered one year after traumatic brain injury restores behavioral function and normalizes 5-HT2A receptor signaling, while reducing microglial density in the prefrontal cortex in rats, highlighting its potential for patients with chronic TBI symptoms.

## Introduction

Traumatic brain injury (TBI) is a pressing global health challenge that affects ∼70 million people annually.^1^^,^^2^ TBI frequently results in persistent cognitive, emotional, and motor dysfunction.^3^^,^^4^ Beyond immediate aftermath of the injury, TBI heightens the risk of development of neuropsychiatric and neurodegenerative disorders.^5^ Despite this burden, no pharmacological interventions have been approved to target long-term outcomes.

Serotonergic (5-HT) dysregulation has emerged as a contributor to emotional and behavioral symptoms after TBI and psychiatric comorbidities.^6^^,^^7^ Recent preclinical studies indicate that blast-induced TBI disrupts cortical 5-HT_2A_ receptor signaling and expression, which correlates with behavioral impairments, while pharmacological modulation of this system can reverse these deficits.^8^^,^^9^ In addition to their neuronal expression, 5-HT_2A_ receptors are present on microglia,^10^ which adopt a sustained reactive state in chronic TBI that drives neuroinflammation and neuronal damage.^11^^,^^12^^,^^13^^,^^14^^,^^15^ This suggests a potential role for 5-HT_2A_ signaling in both neural and immune aspects of TBI pathology.

Psilocybin, a potent 5-HT_2A_ agonist, shows promise in treating complex neuropsychiatric conditions like depression, anxiety, and post-traumatic stress disorder,^16^^,^^17^^,^^18^^,^^19^^,^^20^^,^^21^^,^^22^^,^^23^ which share key pathophysiological features with TBI, including impaired neuroplasticity, neuroinflammation, and neurotransmission.^24^^,^^25^ Psilocybin’s therapeutic effects are thought to involve enhanced neuroplasticity, synaptogenesis, and anti-inflammatory signaling mediated by 5-HT_2A_ receptors.^10^^,^^26^^,^^27^^,^^28^^,^^29^^,^^30^^,^^31^

Given the persistence of neurobehavioral and microglial abnormalities long after injury,^32^^,^^33^ we examined the effects of psilocybin—administered 1-year post-TBI—on behavior, learning and memory, 5-HT_2A_ receptor binding, and microglial density and morphology. This chronic time point was intended to represent a clinical phase where recovery has plateaued and residual deficits remain resistant to existing treatments.

## Results

### Acute TBI effects

**Figure 1:**
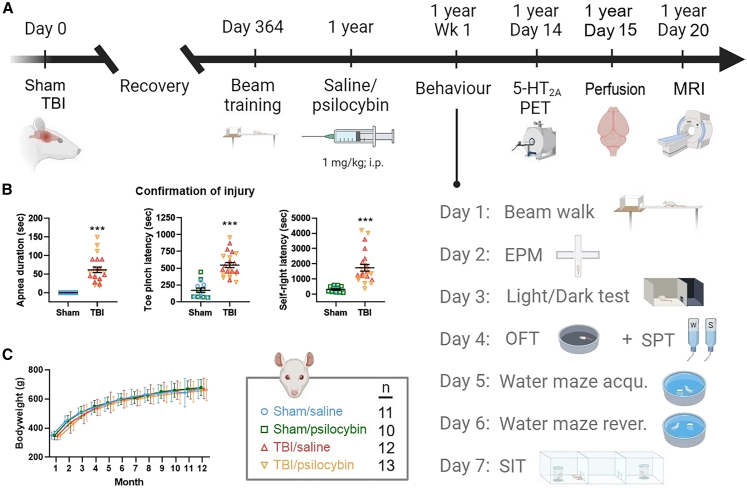
Experimental design (A) Rats were subjected to either sham injury or FPI, followed by a 1-year recovery period. Thereafter, the rats received treatment with either saline or psilocybin, given intraperitoneally at a dose of 1 mg/kg. Twenty-four hours after the treatment, daily behavioral testing occurred for 1 week. Two weeks after the treatment, a subset of rats were subjected to PET scans to assess 5-HT_2A_ receptor binding. The following day, the rats underwent transcardial perfusion. Finally, *ex vivo* MRI scans were performed a week later. (B) TBI extended apnea duration and delayed response to a toe pinch and self-righting (∗∗∗*p* < 0.001, sham vs. TBI; Mann-Whitney U test). (C) TBI had no effect on body weight. PET, positron emission tomography; EPM, elevated plus maze; OFT, open-field test; SPT, sucrose preference test; SIT, social interaction test. Data are presented as the mean ± SEM.

To confirm TBI severity, Mann-Whitney U tests were used to examine apnea duration (U = −5.972, *p* < 0.001), latency to respond to a toe pinch (U = −4.854, *p* < 0.001), and latency to self-right (U = −5.228, *p* < 0.001), all of which were significantly increased following injury (Figure 1B). Notably, Kruskal-Wallis followed by Dunn’s post-hoc analyses showed that there were no significant differences in acute injury measures between the psilocybin or saline treatment groups that were assigned thereafter (apnea duration: H = 35.663, *p* < 0.001, TBI/saline vs. TBI/psilocybin, *p* = 0.957; latency to respond to a toe pinch: H = 24.088, *p* < 0.001, TBI/saline vs. TBI/psilocybin, *p* = 0.492; latency to self-right: H = 27.969, *p* < 0.001, TBI/saline vs. TBI/psilocybin, *p* = 0.426). TBI had no effect on body weight throughout the study (F_(1, 44)_ = 0.052, *p* = 0.820) (Figure 1C), but there was an effect of time (F_(3.835, 168.742)_ = 593.330, *p* < 0.001) without an interaction effect of TBI and time (F_(3.835, 168.742)_ = 0.314, *p* = 0.861), indicating that all groups gained weight at a similar rate.

### The effect of TBI and psilocybin on chronic behavior

**Figure 2:**
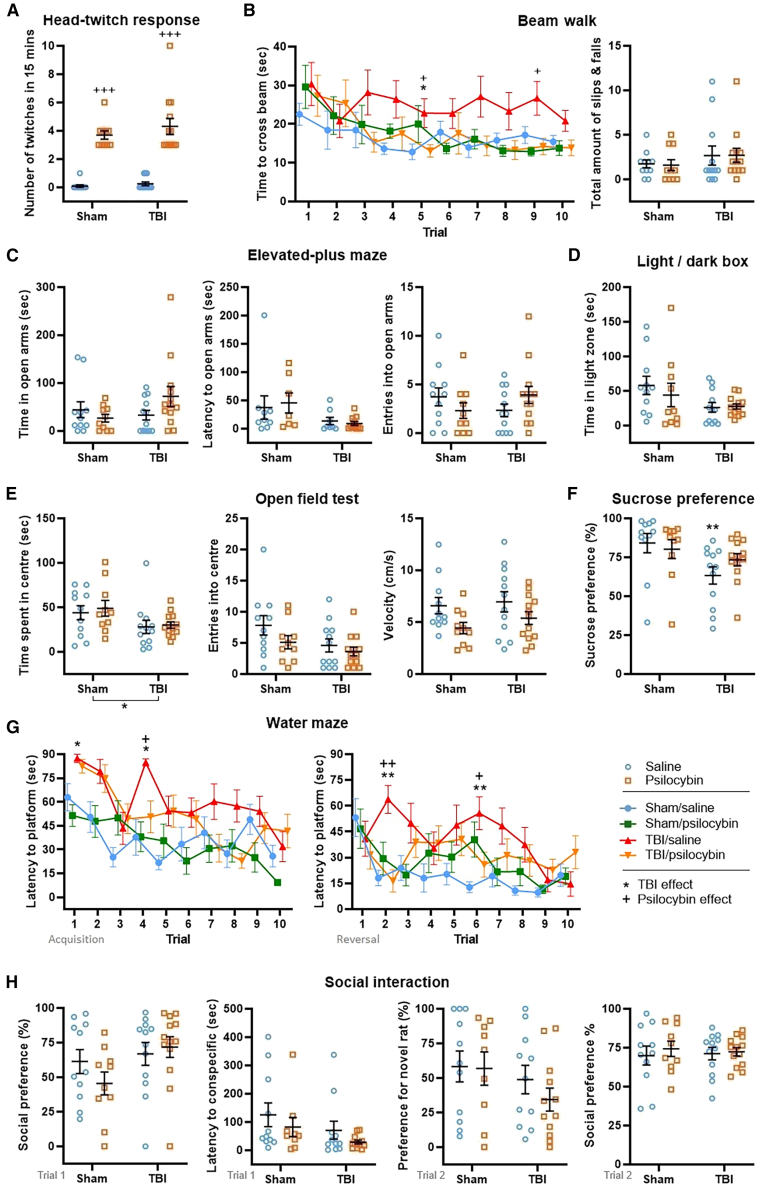
The effect of TBI and psilocybin on behavior (A) Psilocybin increased head-twitching behavior (+++*p* < 0.001, sham/saline vs. sham/psilocybin and TBI/saline vs. TBI/psilocybin; Dunn’s test). (B) TBI impaired sensorimotor function in the beam-walk task, and psilocybin improved the beam-walk task performance (∗*p* < 0.05, sham/saline vs. TBI/saline; +*p* < 0.05, TBI/saline vs. TBI/psilocybin; Dunn’s test). See Figure S1A for beam training data. (C and D) There were no effects of TBI or psilocybin on exploratory behavior in the elevated-plus maze test (C) and light/dark tests (D). (E) TBI reduced time spent in the center of the open-field arena (∗*p* < 0.05 = sham vs. TBI; two-way ANOVA TBI main effect). (F) TBI decreased sucrose preference in sham-treated rats but not psilocybin-treated rats (∗∗*p* < 0.01, sham/saline vs. TBI/saline; Dunn’s test). (G) All rats found the water maze platform faster over time in the acquisition and reversal phases, but saline-treated TBI rats often took longer to find the platform than psilocybin-treated TBI rats and sham-injured rats (∗*p* < 0.05/∗∗*p* < 0.01, sham/saline vs. TBI/saline; +*p* < 0.05/++*p* < 0.01 = TBI/saline vs. TBI/psilocybin; Dunn’s test). See Figures S1B and S1C for swim speed and time spent in target quadrant. (H) There were no effects of TBI or psilocybin on social behavior. Data are presented as the mean ± SEM.

Statistical outcomes for the behavioral data, presented in Figure 2, are summarized in Table 1. Thus, we report detailed statistics only for post-hoc group comparisons. These findings should be interpreted with caution, as the large number of behavioral tests used increases the risk of false positives.

**Table 1:** Statistical information for behavior

Behavioral statistics	TBI effect	Psilocybin effect	Injury × Psilocybin
Head-twitch	Kruskal-Wallis: H = 36.867, ***p* < 0.001∗∗∗**		
Beam-walk trial 1	Kruskal-Wallis: H = 0.912, *p* = 0.823		
Beam-walk trial 2	Kruskal-Wallis: H = 0.927, *p* = 0.819		
Beam-walk trial 3	Kruskal-Wallis: H = 4.289, *p* = 0.232		
Beam-walk trial 4	Kruskal-Wallis: H = 4.289, *p* = 0.232		
Beam-walk trial 5	Kruskal-Wallis: H = 9.048, ***p* = 0.029∗**		
Beam-walk trial 6	Kruskal-Wallis: H = 5.748, *p* = 0.125		
Beam-walk trial 7	Kruskal-Wallis: H = 7.052, *p* = 0.070		
Beam-walk trial 8	Kruskal-Wallis: H = 4.399, *p* = 0.221		
Beam-walk trial 9	Kruskal-Wallis: H = 10.671, ***p* = 0.014∗**		
Beam-walk trial 10	Kruskal-Wallis: H = 5.465, *p* = 0.141		
Beam slips & falls	Kruskal-Wallis: H = 2.383, *p* = 0.497		
EPM time in open	Kruskal-Wallis: H = 4.625, *p* = 0.201		
EPM latency to open	Kruskal-Wallis: H = 6.050, *p* = 0.109		
EPM open entries	Kruskal-Wallis: H = 3.185, *p* = 0.364		
Light/dark test	F_(1, 42)_ = 1.284, *p* = 0.264	F_(1, 42)_ = 0.083, *p* = 0.775	F_(1, 42)_ = 0.648, *p* = 0.425
OFT time in center	F(_1, 42)_ = 6.290, ***p* = 0.016∗**	F_(1, 42)_ = 0.259, *p* = 0.613	F_(1, 42)_ = 0.083, *p* = 0.847
OFT center entries	Kruskal-Wallis: H = 5.890, *p* = 0.117		
OFT velocity	F_(1, 42)_ = 0.783, *p* = 0.381	F_(1, 42)_ = 5.984, ***p* = 0.019∗**	F_(1, 42)_ = 0.143, *p* = 0.708
Sucrose preference	Kruskal-Wallis: H = 13.217, ***p* = 0.004∗∗**		
Water maze acquisition trial 1	Kruskal-Wallis: H = 20.271, ***p* < 0.001∗∗∗**		
Water maze acquisition trial 2	Kruskal-Wallis: H = 8.576, ***p*****= 0.035∗**		
Water maze acquisition trial 3	Kruskal-Wallis: H = 3.607, *p* = 0.307		
Water maze acquisition trial 4	Kruskal-Wallis: H = 12.196, ***p* = 0.007∗∗**		
Water maze acquisition trial 5	Kruskal-Wallis: H = 6.070, *p* = 0.108		
Water maze acquisition trial 6	Kruskal-Wallis: H = 6.035, *p* = 0.110		
Water maze acquisition trial 7	Kruskal-Wallis: H = 4.327, *p* = 0.228		
Water maze acquisition trial 8	Kruskal-Wallis: H = 6.096, *p* = 0.107		
Water maze acquisition trial 9	Kruskal-Wallis: H = 5.797, *p* = 0.122		
Water maze acquisition trial 10	Kruskal-Wallis: H = 3.891, *p* = 0.274		
Water maze reversal trial 1	Kruskal-Wallis: H = 2.407, *p* = 0.492		
Water maze reversal trial 2	Kruskal-Wallis: H = 15.835, ***p* = 0.001∗∗**		
Water maze reversal trial 3	Kruskal-Wallis: H = 6.063, *p* = 0.109		
Water maze reversal trial 4	Kruskal-Wallis: H = 3.049, *p* = 0.384		
Water maze reversal trial 5	Kruskal-Wallis: H = 2.393, *p* = 0.495		
Water maze reversal trial 6	Kruskal-Wallis: H = 12.295, ***p* = 0.006∗∗**		
Water maze reversal trial 7	Kruskal-Wallis: H = 3.860, *p* = 0.277		
Water maze reversal trial 8	Kruskal-Wallis: H = 3.856, *p* = 0.277		
Water maze reversal trial 9	Kruskal-Wallis: H = 5.452, *p* = 0.142		
Water maze reversal trial 10	Kruskal-Wallis: H = 4.777, *p* = 0.189		
SIT social preference T2	F_(1, 42)_ = 3.786, *p* = 0.058	F_(1, 42)_ = 0.442, *p* = 0.510	F_(1, 42)_ = 1.625, *p* = 0.209
SIT latency to rat T2	Kruskal-Wallis: H = 6.447, *p* = 0.092		
SIT preference for novel T3	F_(1, 39)_ = 2.357, *p* = 0.133	F_(1, 39)_ = 0.593, *p* = 0.446	F_(1, 39)_ = 0.384, *p* = 0.539
SIT social preference T3	F_(1, 42)_ = 0.005, *p* = 0.942	F_(1, 42)_ = 0.374, *p* = 0.544	F_(1, 42)_ = 0.126, *p* = 0.725

TBI produced deficits across sensorimotor, behavioral, and cognitive domains, which were attenuated by psilocybin treatment. Significant results are bolded: ∗*p* < 0.05; ∗∗*p* < 0.01; ∗∗∗*p* < 0.001.

### Head-twitch response

Dunn’s post-hoc analyses revealed that psilocybin induced head-twitch responses in sham (*p* < 0.001) and TBI rats (*p* < 0.001; Figure 2A).

### Beam-walk test

Dunn’s post-hoc analyses revealed that TBI/saline rats took longer to cross the beam than sham/saline rats in trial 5 (*p* = 0.010), and TBI/psilocybin rats traversed the beam significantly quicker than TBI/saline rats in trial 5 (*p* = 0.016) and trial 9 (*p* = 0.035; Figure 2B). No effects of TBI or psilocybin on the number of slips and falls were observed.

Beam training data were analyzed to ensure that, within the TBI group, the rats later assigned to psilocybin or saline treatment did not differ prior to drug administration. No differences in beam training performance were observed between the rats later assigned to psilocybin or saline treatment within either the sham or TBI groups (see Table S1; Figure S1A).

### Anxiety tests

There were no main effects of TBI or psilocybin on elevated plus-maze (Figure 2C) and light/dark test (Figure 2D) behaviors. TBI decreased the time spent in the center of the open-field arena, but there were no significant post-hoc group differences (Figure 2E).

### Sucrose preference test

Dunn’s post-hoc analyses revealed that sucrose preference was significantly decreased in TBI/saline rats compared with sham/saline rats (*p* = 0.006; Figure 2F).

### Water maze

Acquisition: Dunn’s post-hoc analyses showed that TBI/saline rats required significantly more time to locate the platform than sham/saline rats in trial 1 (*p* = 0.021) and trial 4 (*p* = 0.020). In contrast, TBI/psilocybin rats found the platform significantly faster than TBI/saline rats in trial 4 (*p* = 0.029; Figure 2G). However, TBI/psilocybin rats took longer in trial 1 than sham/psilocybin rats (*p* = 0.006).

Reversal: Dunn’s post-hoc analyses indicated that TBI/saline rats took significantly longer to find the platform than sham/saline rats in trial 2 (*p* = 0.017) and trial 6 (*p* = 0.005). TBI/psilocybin rats showed significantly faster search times than TBI/saline rats in trial 2 (*p* = 0.001) and trial 6 (*p* = 0.033).

Swim speed and time spent in the target quadrant were assessed to determine whether differences in platform latency reflected learning and memory performance, rather than alterations in locomotor activity, motivation, or search behavior. There were no group differences in swim speed or time spent in the target quadrant (see Table S1; Figures S1B and S1C).

### Social interaction test

There were no main effects of TBI or psilocybin on social behavior (Figure 2H).

### The effect of TBI and psilocybin on 5-HT_2A_ receptor binding

**Figure 3:**
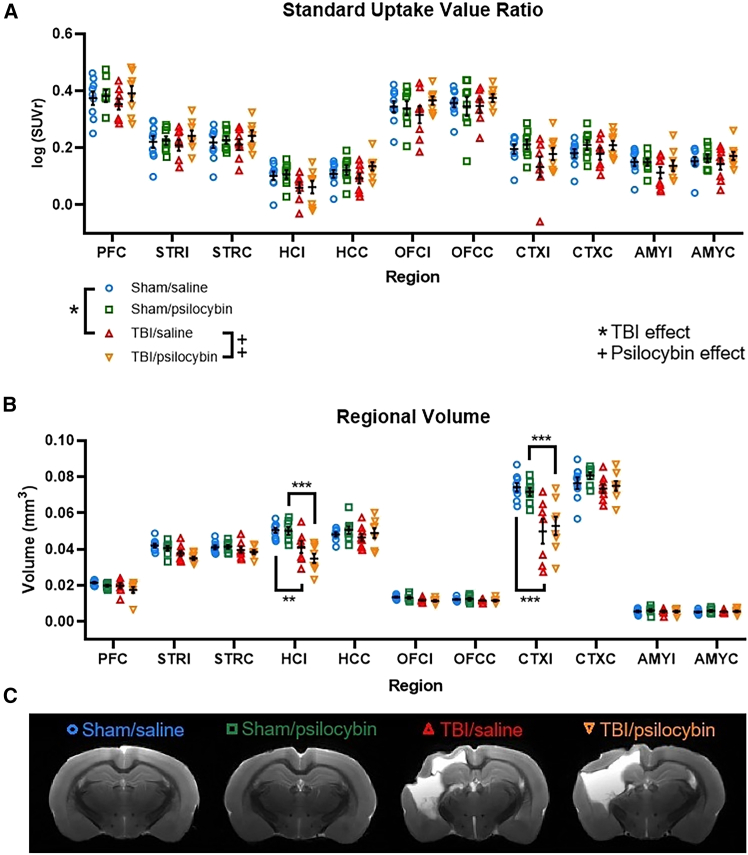
PET 5-HT_2A_ receptor binding density (A) Psilocybin recovered 5-HT_2A_ receptor binding deficits in TBI rats (∗*p* < 0.05, sham/saline vs. TBI/saline; ++*p* < 0.01, TBI/saline vs. TBI/psilocybin; Tukey’s test). (B) TBI was associated with a significant reduction in volume in both the ipsilateral hippocampus and cortex in both sham and psilocybin-treated rats (∗∗*p* < 0.01/∗∗∗*p* < 0.001, sham/saline vs. TBI/saline or sham/psilocybin vs. TBI/psilocybin; Tukey’s test). See Figures S1D and S1E for volume vs. 5-HT_2A_ binding. (C) Representative MRI images illustrating the extent of volume loss following TBI across experimental groups. Data are presented as the mean ± SEM. PFC, prefrontal cortex; STRI/STRC, striatum ipsilateral/contralateral; HCI/HCC, hippocampus ipsilateral/contralateral; OFCI/OFCC, orbitofrontal cortex ipsilateral/contralateral; CTXI/CTXC, motor, sensory, and auditory cortices ipsilateral/contralateral; AMYI/AMYC, amygdala ipsilateral/contralateral.

A significant group effect was observed in the manually defined volumes of interest (VOIs) (F_(3, 315)_ = 5.808, *p* < 0.001). Tukey’s post-hoc tests showed that TBI/saline rats had lower binding than sham/saline (*p* = 0.033), sham/psilocybin (*p* = 0.003), and TBI/psilocybin (*p* = 0.001) rats (Figure 3A), demonstrating that psilocybin recovers TBI-induced reductions in 5-HT_2A_ receptor binding. There was also a significant main effect of brain region on 5-HT_2A_ standardized uptake value ratio (SUVr) (F_(10, 315)_ = 104.900, *p* < 0.001); however, no region-specific effects were observed in the injury/treatment group, and no region × group interaction (F_(30, 315)_ = 0.452, *p* = 0.995) was observed.

To explore these group differences further, voxel-wise analyses were conducted between TBI and sham saline-treated rats and between saline-and psilocybin-treated TBI rats. Exploratory voxel-wise analyses revealed clusters of reduced binding in the perilesional cortex and posterior hippocampus in saline-treated TBI rats compared with sham controls (peak T = 4.05, punc = 0.001; Figure S1D). In contrast, psilocybin-treated TBI rats showed increased binding relative to vehicle-treated rats in regions including the superior colliculus, retrosplenial granular cortex (peak T = 3.76, punc = 0.001), and ipsilateral deep mesencephalic nucleus (peak T = 3.26, punc = 0.003). However, none of these clusters survived correction for multiple comparisons (family-wise error, *p* > 0.05; Figure S1D).

5HT_2A_ binding in the prefrontal cortex (PFC) did not correlate with head-twitch response (R = −0.173, *p* = 0.521, *N* = 16), indicating that regional receptor availability in this region was not directly associated with behavioral sensitivity to psilocybin.

### The effect of TBI on MRI volumetric measures

To rule out injury severity as a confound for psilocybin’s effects on 5-HT_2A_ binding, volumetric differences were assessed using manual VOIs and voxel-wise analysis. Manual VOIs revealed significant main effects of group (F_(3, 311)_ = 22.600, *p* < 0.001), region (F_(10, 311)_ = 624.300, *p* < 0.001), and their interaction (F_(30, 311)_ = 4.422, *p* < 0.001). Both TBI/saline and TBI/psilocybin groups had reduced ipsilateral hippocampus and cortex volumes (*p* ≤ 0.004) compared with their respective sham counterparts, with no differences between the TBI treatment groups (Figures 3B and 3C).

To further confirm that regional volume did not confound positron emission tomography (PET) 5-HT_2A_ binding measures, PET-derived binding density was compared with regional brain volumes in the hippocampus and cortex for the TBI/psilocybin and TBI/saline groups (Figure S1E). In the hippocampus, correlations were weak and non-significant for both TBI/psilocybin (r = 0.0645, *p* = 0.879) and TBI/saline rats (r = 0.0513, *p* = 0.913). Similarly, in the cortex, no significant relationships were observed for TBI/psilocybin (r = 0.004, *p* = 0.993) or TBI/saline rats (r = 0.368, *p* = 0.417), indicating that psilocybin-related differences in 5-HT2A binding were not influenced by regional volume (see Figure S1E).

### The effect of TBI and psilocybin on microglial cell counts and morphology

**Figure 4:**
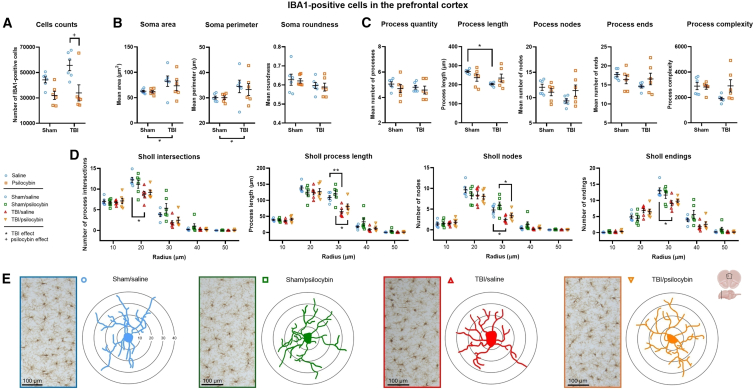
The effect of TBI and psilocybin on microglial cell morphology in the prefrontal cortex (A) Psilocybin reduced the number of IBA1-positive microglial counts, quantified across four tissue sections per rat (+*p* < 0.05, TBI/saline vs. TBI/psilocybin; Tukey’s test). (B–D) TBI (B) increased soma size (∗*p* < 0.05, sham vs. TBI; two-way ANOVA TBI main effect), (C) decreased process length (∗*p* < 0.05, sham/saline vs. TBI/saline; Tukey’s test), and (D) altered Sholl metrics, i.e., intersections, process length, and number of process nodes and endings, all derived from the average of ten traced cells per rat (∗*p* < 0.05/∗∗*p* < 0.01, sham/saline vs. TBI/saline or sham/psilocybin vs. TBI/psilocybin; Tukey’s test). (E) Representative photomicrographs of IBA1-positive microglia and individual tracings for Sholl analyses. Data presented as the mean ± SEM. Scale bars are 100 μm.

Microglial outcomes, assessed via ionized calcium-binding adapter molecule 1 (IBA1) immunohistochemical staining (Figure 4), are summarized in Table 2; below we report the key findings.

**Table 2:** Statistical information for IBA1-positive cells in the PFC

IBA1-positive cells	TBI effect	Psilocybin effect	TBI × Psilocybin
IBA1 cell counts	F_(1, 20)_ = 2.414, *p* = 0.136	F_(1, 20)_ = 15.545, ***p* = 0.001∗∗∗**	F_(1, 20)_ = 1.205, *p* = 0.285
Soma area	F_(1, 20)_ = 4.745, ***p* = 0.042∗**	F_(1, 20)_ = 0.506, *p* = 0.485	F_(1, 20)_ = 0.313, *p* = 0.582
Soma perimeter	F_(1, 20)_ = 4.649, ***p* = 0.043∗**	F_(1, 20)_ = 0.126, *p* = 0.726	F_(1, 20)_ = 0.115, *p* = 0.738
Soma roundness	F_(1, 20)_ = 2.386, *p* = 0.138	F_(1, 20)_ = 0.128, *p* = 0.724	F_(1, 20)_ = 0.002, *p* = 0.962
Process quantity	F_(1, 20)_ = 0.509, *p* = 0.484	F_(1, 20)_ = 1.329, *p* = 0.263	F_(1, 20)_ = 0.075, *p* = 0.787
Process length	F_(1, 20)_ = 5.632, ***p* = 0.028∗**	F_(1, 20)_ = 0.001, *p* = 0.991	F_(1, 20)_ = 5.045, ***p* = 0.036∗**
Process nodes	F_(1, 20)_ = 2.548, *p* = 0.126	F_(1, 20)_ = 0.497, *p* = 0.489	F_(1, 20)_ = 4.018, *p* = 0.059
Process ends	F_(1, 20)_ = 2.156, *p* = 0.158	F_(1, 20)_ = 0.111, *p* = 0.743	F_(1, 20)_ = 2.671, *p* = 0.118
Process complexity	F_(1, 20)_ = 2.242, *p* = 0.150	F_(1, 20)_ = 2.454, *p* = 0.133	F_(1, 20)_ = 3.252, *p* = 0.086
Sholl - intersections 10 μm	F_(1, 20)_ = 0.081, *p* = 0.779	F_(1, 20)_ = 0.003, *p* = 0.995	F_(1, 20)_ = 0.604, *p* = 0.446
Sholl - intersections 20 μm	F_(1, 20)_ = 14.452, ***p* = 0.001∗∗**	F_(1, 20)_ = 0.174, *p* = 0.681	F_(1, 20)_ = 1.088, *p* = 0.309
Sholl - intersections 30 μm	Kruskal-Wallis: H = 9.217, ***p* = 0.027∗**		
Sholl - intersections 40 μm	Kruskal-Wallis: H = 4.887, *p* = 0.180		
Sholl - intersections 50 μm	Kruskal-Wallis: H = 2.094, *p* = 0.553		
Sholl - length 10 μm	F_(1, 20)_ = 0.024, *p* = 0.878	F_(1, 20)_ = 0.969, *p* = 0.337	F_(1, 20)_ = 1.329, *p* = 0.263
Sholl - length 20 μm	F_(1, 20)_ = 0.357, *p* = 0.557	F_(1, 20)_ = 0.580, *p* = 0.445	F_(1, 20)_ = 1.319, *p* = 0.264
Sholl - length 30 μm	F_(1, 20)_ = 27.775, ***p* < 0.001∗∗∗**	F_(1, 20)_ = 2.881, *p* = 0.105	F_(1, 20)_ = 0.776, *p* = 0.766
Sholl - length 40 μm	Kruskal-Wallis: H = 8.367, ***p* = 0.039∗**		
Sholl - length 50 μm	Kruskal-Wallis: H = 4.672, *p* = 0.197		
Sholl - nodes 10 μm	F_(1, 20)_ = 0.322, *p* = 0.577	F_(1, 20)_ = 0.589, *p* = 0.452	F_(1, 20)_ = 0.097, *p* = 0.759
Sholl - nodes 20 μm	F_(1, 20)_ = 1.147, *p* = 0.297	F_(1, 20)_ = 1.295, *p* = 0.269	F_(1, 20)_ = 0.479, *p* = 0.497
Sholl - nodes 30 μm	F_(1, 20)_ = 17.135, ***p* = 0.001∗∗**	F_(1, 20)_ = 3.089, *p* = 0.094	F_(1, 20)_ = 0.017, *p* = 0.897
Sholl - nodes 40 μm	Kruskal-Wallis: H = 8.454, ***p* = 0.038∗**		
Sholl - nodes 50 μm	Kruskal-Wallis: H = 2.488, *p* = 0.477		
Sholl - endings 10 μm	Kruskal-Wallis: H = 5.282, *p* = 0.152		
Sholl - endings 20 μm	F_(1, 20)_ = 8.600, ***p* = 0.008∗∗**	F_(1, 20)_ = 0.289, *p* = 0.597	F_(1, 20)_ = 0.015, *p* = 0.904
Sholl - endings 30 μm	F_(1, 20)_ = 12.595, ***p* = 0.002∗∗**	F_(1, 20)_ = 0.165, *p* = 0.689	F_(1, 20)_ = 1.530, *p* = 0.230
Sholl - endings 40 μm	Kruskal-Wallis: H = 9.837, ***p* = 0.020∗**		
Sholl - endings 50 μm	Kruskal-Wallis: H = 5.312, *p* = 0.150		

TBI altered microglial cell morphology, and psilocybin reduced microglial cell counts. Significant results are bolded: ∗*p* < 0.05; ∗∗*p* < 0.01; ∗∗∗*p* < 0.001.

Psilocybin treatment significantly decreased IBA1-positive microglial count, with Tukey’s post-hoc analyses indicating that TBI/psilocybin rats had significantly fewer cells than TBI/saline rats (*p* = 0.010; Figure 4A).

TBI significantly increased microglial soma area and perimeter but not roundness. No main effects of psilocybin or TBI × psilocybin interactions were detected, and there were no significant Tukey post-hoc differences (Figure 4B).

Tukey’s post-hoc tests revealed that sham/saline rats had significantly longer processes than TBI/saline rats (*p* = 0.019; Figure 4C).

Tukey’s post-hoc tests revealed that, compared with sham/saline rats, TBI/saline rats had fewer intersections at 20 μm (*p* = 0.013) and shorter processes (*p* = 0.004), fewer nodes (*p* = 0.046), and fewer endings (*p* = 0.014) at 30 μm (Figure 4D). Additionally, compared with sham/psilocybin rats, TBI/psilocybin rats had shorter process lengths (*p* = 0.011) and fewer nodes (*p* = 0.032) at 30 μm.

## Discussion

This study assessed the therapeutic effects of a single psilocybin dose in mitigating behavioral, neurobiological, and neuroimaging deficits 1-year post-TBI. Our findings demonstrate that: (1) psilocybin reversed motor and learning and memory deficits; (2) psilocybin restored 5-HT_2A_ receptor binding; and (3) psilocybin decreased microglial density in the PFC.

The potential therapeutic effects of psilocybin were evaluated in a well-established fluid-percussion TPI (FPI) model,^34^^,^^35^ mimicking a clinically relevant scenario in which treatment is often sought long after the typical window for spontaneous recovery. Behavioral testing revealed that psilocybin improved sensorimotor functioning in the beam-walk test. Additional research should evaluate whether this effect is driven by enhanced synaptic plasticity, neurogenesis, or serotonergic modulation within motor and somatosensory pathways. TBI rats treated with psilocybin had statistically significant faster search times in 2 of 10 trials during water maze reversal compared with their saline-treated counterparts. However, given the restricted nature of these findings, their biological significance and whether they constitute a cognitive improvement remain uncertain and require further investigation. For example, it would be interesting to examine whether psilocybin’s effects are linked to enhanced cognitive flexibility, which would align with clinical observations in depressed patients.^36^ Psilocybin has been shown to restore sucrose preference in other models.^26^^,^^37^^,^^38^ Although a modest increase in sucrose preference was produced by psilocybin in this study, this effect did not reach statistical significance. Taken together, these findings underscore psilocybin’s potential to mitigate some, but not all, chronic TBI symptoms. Nevertheless, we acknowledge the inherent limitations of behavioral testing in animal research, particularly the interpretive challenges associated with subjectivity and anthropomorphic biases.

The 5-HT_2A_ receptor plays a pivotal role in both physiological and pathological brain functions and represents a compelling target for neurorehabilitation following TBI.^8^^,^^39^^,^^40^^,^^41^ In the present study, psilocybin elicited a robust head-twitch response, a well-established behavioral proxy of 5-HT_2A_ receptor activation, and normalized TBI-induced deficits in 5-HT_2A_ receptor binding. Psilocybin had no effect on 5-HT_2A_ binding in sham rats, suggesting minimal receptor-level changes in healthy brains, and supporting the interpretation that its effects may be context dependent, emerging primarily under conditions of injury or altered neural function. The absence of TBI effects on head-twitch behavior suggests that, despite lower receptor availability, sufficient 5-HT_2A_ signaling capacity remained to generate a comparable response to sham injured rats.

Psilocybin may restore receptor density through balanced internalization and recycling^42^^,^^43^ and enhanced protein synthesis via 5-HT_2A_-G_q_-coupled and tropomyosin receptor kinase B (TrkB) pathways,^29^^,^^44^^,^^45^ thereby stabilizing neurotransmission to support recovery. Furthermore, psychedelics may also induce neuroplasticity and regulate receptor dynamics by targeting intracellular 5-HT_2A_ receptors, activating pathways not typically engaged by endogenous 5-HT.^30^ Discrepancies between our findings and prior reports of increased 5-HT_2A_ binding in the frontal cortex shortly after blast-induced mild TBI^8^ likely reflect differences in injury mechanism, the comparison of acute versus chronic time points, and radioligand properties. Specifically, [^3^H]ketanserin used in earlier work exhibits lower selectivity for 5-HT_2A_ receptors and binds to α_1_-adrenergic and histaminergic sites, potentially inflating non-specific binding, whereas [^18^F]altanserin used here exhibits greater affinity and improved specificity for 5-HT_2A_ receptors.^8^^,^^46^^,^^47^^,^^48^ Variations in pharmacological properties between these ligands, including brain penetration and metabolic stability, could, therefore, account for the divergent receptor availability observed across studies.^49^^,^^50^

Post-injury, microglia can adopt maladaptive phenotypes that perpetuate chronic pathology, as seen in human brain tissue showing persistent activation years later.^32^ Given the PFC’s central role in higher-order cognitive function and sensorimotor integration, its vulnerability to TBI-related neuroinflammation,^51^^,^^52^ and its high density of 5-HT_2A_ receptors, we examined whether psilocybin’s behavioral effects correspond with changes in microglial density and morphology within this region. We found that TBI reduced microglial complexity, evidenced by shorter process lengths and fewer Sholl intersections, branching nodes, and terminal endings within 20–40 μm of the soma. While psilocybin did not reverse these morphological changes, it lowered the cell density. Psilocybin treatment has also been shown to reduce microglial density in the hippocampus following brain injury in a female rat model of intimate partner violence-related brain injury.^53^ Excessive or prolonged microglial activation can exacerbate synaptic dysfunction and neuroinflammation; so, normalization of microglial populations may promote a more permissive environment for synaptic plasticity and circuit recovery. This was demonstrated in a mild FPI model, where depleting microglia attenuated chronic neuroinflammation, restored cortical dendritic architecture, and improved cognitive outcomes,^14^ which underscore their role in long-term neurodegeneration. Microglial changes may be linked to the upregulation of 5-HT_2A_ receptor binding, as interactions between 5-HT signaling and microglial reactivity are increasingly recognized.^10^^,^^54^^,^^55^ Supporting this, greater 5-HT_2A_ receptor binding in the PFC of untreated TBI rats was associated with smaller soma and longer processes. Psilocybin’s effects may also involve indirect enhancement of the release of endogenous 5-HT and other neuromodulators through mechanisms downstream of 5-HT_2A_ receptors, such as glutamate release and neurotrophic signaling.^44^^,^^56^^,^^57^

The necessity of 5-HT_2A_ activation in psilocybin’s therapeutic effects remains uncertain. While 5-HT_2A_ antagonists block the head-twitch response in rodents, they do not appear to inhibit psilocybin’s antidepressant-like, electrophysiological, or neuroplastic effects *in vivo.*^27^^,^^38^ Still, 5-HT_2A_-driven glutamatergic surges promote synaptic strengthening^38^^,^^58^ and neuroplasticity,^27^^,^^29^^,^^44^^,^^59^ likely contributing to therapeutic outcomes. This is supported by *in vitro* studies, which demonstrated that psychedelic-induced structural plasticity is abolished not only by 5-HT_2A_ antagonism but also by inhibition of TrkB, mTOR, and AMPA receptors, key mediators of neuronal growth, survival, and excitability.^29^^,^^59^ Moreover, psilocybin upregulates brain-derived neurotrophic factor (BDNF) and allosterically modulates TrkB receptors, supporting its neuroprotective and neuroregenerative potential.^29^^,^^44^ Recent evidence adds nuance to this framework by demonstrating that psilocybin’s long-term actions can arise through distinct 5-HT_2A_-dependent mechanisms across different cortical circuits. In the mouse medial frontal cortex, psilocybin enhanced dendritic spine density in both the subcortical-projecting pyramidal tract and intratelencephalic cell types; silencing the pyramidal tract neurons prevents psilocybin from reducing stress-related behaviors, while silencing intratelencephalic neurons produces no observable effect.^60^ Psychedelic-induced synaptic potentiation has also been observed in retrosplenial cortex neurons that lack postsynaptic 5-HT_2A_ receptors, an effect driven instead by presynaptic 5-HT_2A_ receptors on thalamic inputs.^61^ These findings suggest that psychedelic-induced plasticity may not require postsynaptic 5-HT_2A_ activation per se but can instead emerge from presynaptic modulation within connected circuits. Given this complexity, testing whether a selective 5-HT_2A_ antagonist such as volinanserin can block psilocybin’s effects in a TBI model would provide insight into receptor-dependent versus receptor-independent mechanisms underlying its therapeutic actions.

In conclusion, psilocybin showed therapeutic potential for chronic TBI, improving behavior, as well as restoring 5-HT_2A_ receptor binding and normalizing the microglial density. These findings warrant further exploration of psilocybin as a promising avenue for TBI treatment and psilocybin’s therapeutic mechanisms of action.

### Limitations of the study

Several limitations should be considered when interpreting these findings. First, our microglial analyses were restricted to the medial PFC. While psilocybin reduced microglial density in this region, these findings should not be interpreted as direct evidence of anti-inflammatory effects. Future studies should include additional brain regions proximal to the injury and incorporate molecular and cellular markers (e.g., cytokines and astrocytic responses) to provide a more comprehensive assessment of neuroimmune modulation.

Similarly, manual VOI analyses focused only on the cortical and limbic regions selected *a priori* based on their established relevance to TBI and psilocybin’s mechanisms of action. Although exploratory voxel-wise analyses revealed subthreshold clusters that did not survive correction for multiple comparisons, future studies with larger sample sizes may improve the statistical power of voxel-wise approaches to detect subtle group differences across the whole brain, including subcortical nuclei such as the thalamus and midbrain.^62^^,^^63^ Furthermore, while this work examined 5-HT_2A_ receptor binding, molecular pathways implicated in psilocybin’s neuronal and neuroimmune interactions (e.g., BDNF, mTOR, and TrkB signaling) were not assessed, limiting mechanistic interpretation. Clarifying these pathways could help disentangle psilocybin’s therapeutic mechanisms from its hallucinogenic effects, advancing future drug development. Neuronal regeneration and astrocytic responses were also not evaluated, though both are likely to contribute to psilocybin-mediated recovery.

The present study employed a single-dose design at a chronic recovery time, which may limit the interpretation of psilocybin’s temporal effects on neuroinflammation and recovery and does not align with some clinical practices that utilize repeated dosing. However, our approach was intentionally selected as a proof-of-concept investigation in a condition not yet explored with psilocybin. Given the dynamic and evolving pathophysiology that occurs during the acute and subacute stages of TBI, initiating work in a chronic model provided a more stable framework to isolate treatment effects and establish foundational evidence of therapeutic potential. While evolving clinical psychedelic therapy paradigms now often involve multiple dosing sessions with varying doses and psychological support,^17^^,^^22^ single-dose designs have been utilized in early clinical trials^64^^,^^65^^,^^66^ and remain relevant for establishing translational groundwork. Future studies incorporating repeated dosing and dose-response relationships (e.g., preclinical doses equivalent to higher doses used in clinical trials), along with other acute, subacute, and chronic post-TBI treatment and assessment times (e.g., treatment within hours, weeks, and months), will be important for mapping psilocybin’s therapeutic window and optimal treatment strategy for TBI.

Replication in female cohorts exposed to FPI is also necessary to assess sex-specific and generalizable effects, given the sexual dimorphism observed in acute and chronic TBI.^67^^,^^68^ Promisingly, a recent study did find benefits for psilocybin treatment in a female rat model of intimate partner violence-related brain injury (i.e., repeated concussion and strangulation), which is a TBI subtype that is particularly common in female humans.^53^

Although this study provides preliminary preclinical evidence of psilocybin-related effects in a TBI model, these limitations restrict its direct clinical relevance and translational utility. The observed behavioral and neurobiological changes cannot be assumed to translate into meaningful functional improvements or clinically relevant recovery outcomes in patients.

## Resource availability

### Lead contact

Requests for further information and resources should be directed to and will be fulfilled by the lead contact, Sandy Shultz (sandy.shultz@monash.edu).

### Materials availability

The radioligand generated in this study cannot be distributed due to its radioactive nature, short half-life, and regulatory constraints governing production, handling, and transport. Detailed synthesis and handling protocols are provided in STAR Methods to enable reproduction at suitably equipped facilities. All other materials used in this study are commercially available and are listed in the key resources table.

### Data and code availability

• The data reported in this study are available from the corresponding author upon reasonable request.
• This paper does not report original code.
• Any additional information required to reanalyze the data reported in this work is available from the lead contact upon request.

## Acknowledgments

The authors thank the USONA Institute Investigational Drug Supply Program for providing psilocybin for this work. The authors acknowledge the facilities and scientific and technical assistance of the National Imaging Facility (NIF), a National Collaborative Research Infrastructure Strategy (NCRIS) capability at Monash Biomedical Imaging (MBI), a Technology Research Platform at Monash University. T.J.O. received funding from the Australian NHMRC, and S.R.S. received funding from 10.13039/501100000245Michael Smith Health Research BC.

## Author contributions

J.A. conducted the experiments, analyzed data, wrote the manuscript draft, and created the figures; B.J. performed the PET scans and analyses; M.B.H. developed the PET radiotracer; R.B. performed the MRI scans; T.L.B., Z.P., M.S., and J.B. assisted with experimental work and data collection; B.R.C., S.J.M., C.T.D., T.J.O., P.M.C.-E., and S.R.S. contributed to data interpretation; P.M.C.-E. and S.R.S. conceptualized the study and experimental design. All authors contributed to manuscript revision and approved the final version.

## Declaration of interests

The authors declare no competing interests.

## STAR★Methods

### Key resources table

**Table:** 

REAGENT or RESOURCE	SOURCE	IDENTIFIER
**Antibodies**		

Rabbit anti-IBA1 primary antibody	Abcam	Cat# ab178846; RRID:AB_2636859
Goat anti-rabbit secondary antibody	Abcam	Cat# ab6720; RRID:AB_954902

**Chemicals, peptides, and recombinant proteins**		

Vectastain ABC complex	Vector Labs	Cat# PK-4000
Hydrogen Peroxide	Millipore Sigma	Cat# 216763-500ML
Triton X-100	Millipore Sigma	Cat# 9036-19-5
Bovine Serum Albumin	Millipore Sigma	Cat# A4737
Normal goat serum	Abcam	Cat# ab7481
DAB	Millipore Sigma	Cat# D4293
Permount Mounting Medium	Fisher Scientific	Cat# SP15-500
[18F]altanserin	Generated by authors at The Peter MacCallum Cancer Center	N/A
nitroaltanserin	Advanced Biochemical Compounds	Cat# 1800

**Experimental models: Organisms/strains**		

Male Sprague-Dawley rats	AMREP animal services Melbourne	N/A

**Software and algorithms**		

TopScan Version 3.0	CleverSys Inc	https://cleversysinc.com/CleverSysInc/csi_products/topscan-lite/
MATLAB Version 2018b	MathWorks	https://www.mathworks.com/products/compiler/matlab-runtime.html
Prism Version 8	GraphPad	https://www.graphpad.com/features
IBM SPSS Version 27	https://www.ibm.com/support/pages/downloading-ibm-spss-statistics-27	N/A
Stereo Investigator	Microbrightfield	https://www.mbfbioscience.com/products/stereo-investigator
Neurolucida	Microbrightfield	https://www.mbfbioscience.com/products/neurolucida/
NeuroExplorer	Microbrightfield	https://www.mbfbioscience.com/products/neurolucida-explorer
3D-OSEM algorithm	Mediso	Tera-Tomo 3D
SPM12	Welcome Trust Center for Neuroimaging	https://www.fil.ion.ucl.ac.uk/spm/software/spm12/
SAMIT toolbox Version 1.3	GitHub	https://mic-umcg.github.io/samit/

**Other**		

Fluid-Percussion Injury device	AmScien instruments	Model FP301 Signal Conditioner
Olympus BX51 microscope	Microscope Central	https://microscopecentral.com/products/olympus-bx51-fluorescence-microscope
nanoScan PET/CT	Mediso	https://mediso.com/global/en/product/pre-clinical-products/nanoscanr-petct
9.4 T Bruker MRI animal scanner	Bruker	N/A
PMOD Version 4.4	Bruker	https://www.bruker.com/en/products-and-solutions/preclinical-imaging/pmod.html
FlexLab	iPHASE technologies	www.iphase.com.au
GE PETtrace cyclotron	GE HealthCare	N/A

### Experimental model and study participant details

#### Animal husbandry

Ten-week-old male Sprague-Dawley rats (*N* = 58) were obtained from AMREP Animal Services (Melbourne, Australia) and pair-housed with *ad libitum* food and water on a 12-h light/dark cycle. After 1 week of habituation and handling, experiments began. All procedures were approved by the AMREP Animal Ethics Committee (E/8166/2021/M) and complied with ARRIVE 2.0^69^ and NHMRC guidelines.

#### Fluid-percussion injury

Figure 1A illustrates the experimental timeline and group allocation. Rats were randomly assigned to receive a fluid-percussion TBI (FPI) or a sham injury.^70^^,^^71^^,^^72^ Following subcutaneous buprenorphine (0.05 mg/kg), anesthesia was induced with 5% isoflurane and maintained at 2–3%. Then, a 5 mm craniotomy (4.5 mm posterior, 2.5 mm left of bregma) was performed under aseptic conditions,^73^ which was then sealed with a plastic cap, cyanoacrylate, and dental cement. The rat was then removed from the nose cone and secured to the FPI device (AmScien Instruments, Richmond, VA, USA) using the head cap, and TBI was delivered via a saline fluid pulse (range 2.46–3.5 atm, average 2.92 atm; equivalent to a clinical severe TBI grading^74^) at first hindlimb withdrawal; sham rats underwent the same procedure without the pulse. Because all rats exhibited apnea following injury, supplemental oxygen (0.5 mL/min; Mediquip Pvt Ltd., Australia) was administered via a nose cone when apnea duration exceeded 10 s. Injury severity was assessed by apnea duration, reflex latency, and self-righting time. Rats then recovered for 1 year.

Twelve rats were excluded due to immediate mortality or poor recovery. The final injury group sizes were: sham (*n* = 21) and FPI (*n* = 25).

### Method details

#### Psilocybin administration

After recovery from TBI, sham and FPI rats were weight-matched and randomly assigned to receive a single intraperitoneal injection of psilocybin (1 mg/kg in saline at a volume of 1 mg/mL; approximating a clinical dose of ∼10–15 mg^75^^,^^76^) or saline control, 24 h before behavioral testing. This dose and timing were selected based on prior evidence of psilocybin’s pro-plasticity and behavioral effects within 24 h of administration,^38^ while avoiding behavioral testing in the acute psychedelic phase. Immediately after treatment, each rat was individually placed into a bedded cage, and the number of head twitches – a well-established 5-HT_2A_ receptor-dependent response that typically peaks within 6–8 min^27^^,^^77^ – were counted for 15 min.^38^ After 1 h, rats were returned to their home cage. Subsequent behavioral, neurobiological, and neuroimaging assessments were performed under blinded conditions.

#### Behavioral testing

Tests were performed in dedicated procedure rooms using automated video tracking software (TopScan 3.0; CleverSys., USA) to reduce experimenter bias.

#### Beam walk test

Sensorimotor function was assessed using a beam-walk task.^70^^,^^78^ Rats were trained the day before treatment with five trials on a 100 × 4 cm beam, followed by five on a 100 × 2 cm beam. Testing occurred 24 h post-treatment with 10 trials on the 100 × 2 cm beam. A maximum time of 60 s was given per trial and rats that fell were assigned a time of 60 s for that trial.

#### Elevated-plus maze

This test assessed anxiety-like or impulsive behavior.^70^ Rats are positioned at the center of a “+”-shaped maze with two opposite arms enclosed by 30 cm walls, and two open arms. Starting facing an open arm, they explore for 5 min. Time spent and entries into each arm were recorded as measures of anxiety.

#### Light/dark test

This test assessed anxiety-like behavior based on aversion to bright spaces.^79^^,^^80^ The arena (30 × 50 × 25 cm) consisted of a brightly lit white chamber (>200 lux; two-thirds) and a dim black chamber (<10 lux; one-third), connected by a 7 × 7 cm opening. Rats were placed in the light chamber and allowed to explore for 5 min. More time in the dark chamber indicated greater anxiety.

#### Open-field test

This test assessed exploratory and anxiety-like behavior.^70^ Rats were placed into the center of a well-lit, circular arena (100 cm diameter) and observed for 5 min. Less time spent in the central zone (66 cm diameter) indicates increased anxiety.

#### Sucrose preference test

This test assessed hedonic behavior.^38^ Rats were first habituated for 24 h with two water bottles, followed by 24 h with two bottles containing 1% sucrose (2- and 3-day post-treatment, respectively). The actual test was conducted 4 days after treatment and lasted 24 h. During testing, a cage divider ensured that each rat had access to one bottle of water and one bottle of sucrose. To control for side preference, bottle positions were counterbalanced and switched after 12 h. Sucrose preference was calculated as the percentage of sucrose intake relative to total fluid consumed, with lower preference indicating anhedonia-like behavior.

#### Water maze

Spatial learning and memory was assessed using a 2-day water maze task.^78^^,^^79^^,^^81^ Rats navigated a black circular pool (163 cm diameter; 26°C–28°C) with a hidden platform submerged 2 cm below the water’s surface and four external visual cues. On day 1 (acquisition), rats were given 10 trials to locate the platform, starting from randomized points (north, south, east, and west). Timing ended when the rat reached the platform or after 90 s. If unsuccessful, the rat was guided to the platform and stayed for 30 s. On day 2 (reversal), the platform was relocated to the opposite quadrant. Latency to reach the platform was used as the cognitive measure.

#### Social interaction test

This test assessed sociability.^79^^,^^82^ Using age- and weight-matched stimulus rats, testing occurred in a transparent three-chambered acrylic arena (100 × 100 × 50 cm), with metal cages in the outer chambers preventing physical contact. The test had three 10-min phases: habituation – where the test rat explored the empty arena; trial 1 – social preference, with one stimulus rat placed in a cage; and trial 2 – social novelty, where a second, novel rat was introduced in the opposite chamber. Time spent in each chamber and near the stimulus cages was recorded.

#### [^18^F]altanserin radiochemical synthesis

Two weeks post-treatment, positron emission tomography (PET) scans assessed 5-HT_2A_ receptor binding using the radioligand [^18^F]altanserin, synthesized through a method adapted from previously published protocols.^83^ Automated production was conducted on an iPHASE FlexLab module using nitroaltanserin (Advanced Biochemical Compounds, Germany) through nucleophilic aromatic substitution with the fluoride-18 (^18^F) ion. [^18^F]Fluoride was produced on a GE PETtrace cyclotron (GE Healthcare, Waukesha, WI, USA) via the 18O(p,n)^18^F nuclear reaction. ^18^F was trapped on a Waters Accell plus light QMA cartridge that was preconditioned with 5 mL of 0.05 molar of K_2_CO_3_ solution followed by 5 mL of water. ^18^F was then eluted directly into the reactor using 1 mL of a solution containing K_2_CO_3_ (3 mg) and Kryptofix 222 (8 mg) in water:acetonitrile mixture (2.5:7.5). The resulting K_222_.K^18^F^−^ mixture was azetropically dried.^84^ The reactor was then cooled to 40°C and 4–5 mg of nitroaltanserin in 1 mL of anhydrous DMSO was added to the dried K_222_.K^18^F^−^. The reaction mixture was heated to 150°C and stirred for 10 minutes. The reactor was cooled to 40°C before the addition of 3 mL of preparative HPLC solvent. The crude reaction mixture was purified using a Phenomenex Kinetex C18 AXIA column (100 Å, 5 μm, 150 × 10 mm). The mobile phase consisted of methanol (MeOH), tetrahydrofuran (THF), and sodium acetate (NaOAc) at a concentration of 0.05 N and pH 5, in a volume ratio of 27:18:55. The elution was performed at a flow rate of 4 mL/min. The fraction containing [^18^F]altanserin (eluting at about 13 min) was then collected, diluted with water (40 mL) and trapped on a C18 SPE cartridge. Finally, [^18^F]altanserin was eluted using ethanol (1 mL) and diluted in saline (10 mL) to afford the desired product in 10–15% radiochemical yield non decay corrected with molar activities ranging from 111 to 185 GBq/umol.

#### PET acquisition

PET imaging was conducted in a subset of rats (Sham/saline: *n* = 9, TBI/saline: *n* = 8, TBI/psilocybin: *n* = 8; weight range: 560-810g) 1 week following the final behavioral assessment. Rats were anesthetized with isoflurane (induction: 5%; maintenance: 1.5–2% in 1 L/min oxygen) and respiratory rate and temperature monitored throughout the scan, with appropriate adjustment to keep within physiological range (50–60 breaths per minute, 37°C) as required. The 5-HT_2A_ receptor binding radiotracer, [^18^F]altanserin, was administered intravenously in a bolus via the dorsal penile vein (activity: 10-25MBq, mass: 0.2–7.7nmol/kg, to coincide with the start of scanning. There were no significant differences in total activity or mass injected between the experimental groups. Dynamic PET scans were acquired in list mode on a small animal nanoScan PET/CT (Mediso Ltd, Budapest, Hungary) immediately following tracer administration, for 144 min. An X-ray CT scan was obtained immediately following each PET scan for attenuation correction. Following PET/CT scans, rats were perfused and brains extracted for MRI scanning.

#### MRI acquisition

MRI was performed using a 9.4 T Bruker instrument and actively decoupled volume transmit resonator and 4- channel surface receive-only coil. T2-weighted images were acquired using the TurboRARE sequence with TR = 2800 ms; TE = 33 ms; averages = 3; FOV = 35 × 35 mm; matrix = 256 × 256; resolution = 137 × 137 μm; slice thickness = 0.8 mm; number of slices = 35.

#### PET image analysis

PET data were rebinned into 4 × 5s, 4 × 10s, 2 × 30s, 2 × 60s, 2 × 300s, 12 × 600s, 1 × 900s frames and reconstructed using a 3D-OSEM algorithm (Tera-Tomo 3D, Mediso) with 6 iterations and 2 subsets, an isotropic 0.6mm voxel resolution, and a matrix of 142 × 142 × 61. Corrections for scatter, attenuation, and decay were applied, and images were calibrated in kBq/ml.

CT scans were manually registered to corresponding T2 weighted MRI and the transformation matrix applied to PET images in PMOD (Bruker, Switzerland). Volumes of interest (VOIs) were manually delineated on each MRI, targeting the PFC, ipsilateral and contralateral orbitofrontal cortex, perilesional cortex, hippocampus, amygdala, and a region encompassing the 9^th^ and 10^th^ cerebellar lobules (referred to as cerebellum). These VOIs were applied to SUVr maps generated using the signal averaged across the 60-90-min frames using the cerebellum as the ref.^85^ allowing receptor binding and regional volume to be quantified within the same anatomical volumes for each subject. Binding measures were subsequently compared to regional volumes to confirm that group differences in 5HT_2A_ availability were not driven by local volume loss.

Exploratory voxelwise analyses, performed using SPM12 (Wellcome Trust Center for Neuroimaging) and the SAMIT toolbox,^86^ were conducted using SUVr maps. A study specific MRI template was generated, and each MRI individually automatically registered to this template using SPM12. Transformation matrices were then applied to brain masked SUVr maps and resultant images Gaussian smoothed using a 0.8 mm^3^ FWHM kernel.

#### Tissue preparation and immunohistochemical analyses

Rats were transcardially perfused with ∼500 mL of ice-cold 0.1 M phosphate buffer (PB; pH 7.4), followed by ∼500 mL of 4% paraformaldehyde in PB. Brains were post-fixed in 4% paraformaldehyde for 48 h at 4°C, then cryoprotected in 30% sucrose for 72 h and flash frozen. Coronal sections (30 μm) were cut on a cryostat (Vibratome ULTRAPRO 5000).

Microglial cells were assessed by immunohistochemical staining for IBA1, a marker of microglial activation and morphology. Free-floating sections were incubated with rabbit anti-IBA1 (1:1000; Abcam, ab178846) in blocking solution (10% goat serum, 0.1% Triton X-100, 1% Bovine Serum Albumin in 0.1 M TBS) for 24 h, followed by biotinylated goat anti-rabbit secondary antibody (1:500; Abcam, ab6720) for 2 h, and ABC complex (1:500; Vector) for 1 h. Immunolabeling was visualized with DAB (0.02%) and hydrogen peroxide (0.0078%). Sections were mounted, air-dried, dehydrated in ethanol, and coverslipped with Permount (Fisher Scientific). Omission of the primary antibody eliminated immunoreactivity.

#### Microglial cell counts and morphological analyses

IBA1-positive microglia were quantified in the medial PFC, a 5-HT_2A_-dense region known to be particularly vulnerable to TBI.^87^^,^^88^^,^^89^ Quantification was performed using an Olympus BX51 microscope and Stereo Investigator software (Microbrightfield). Four sections per brain were analyzed at 40× magnification using unbiased stereology with a modified optical fractionator. Total cell count (N_total_) was calculated using the formula:

$$N_{\text{total}}:\Sigma Q-\times 1\;/\;ssf\times A\left(x,y\;step\right)\;/\;a\left(frame\right)\times t/h\text{,}$$

where ΣQ− is the cell count; ssf is the section sampling fraction (1/6); A(x,y step) is the area associated with each x,y movement (90000 μm^2^); a(frame) is the area of the counting frame (7500 μm^2^); t is the average section thickness; and h = dissector height (20 μm), with a 4 μm guard zone applied.

Microglial morphology was examined using Neurolucida software (Microbrightfield) by tracing cells at a magnification of 100X with oil immersion. Ten randomly selected cells per rat were traced to be consistent with a previous study.^90^ NeuroExplorer (MicroBrightfield) was used to analyze soma and process features. Sholl analyses quantified branching architecture using concentric 10 μm rings centered on the soma, allowing quantification of process intersections, branching nodes, process endings, and the cumulative process length.

### Quantification and statistical analyses

The primary analyses focused on behavioral, neurobiological, and neuroimaging outcomes to test the main effects of psilocybin treatment following TBI. Data were analyzed using SPSS V27, GraphPad prism V8.0.1, and SPM12. Post-hoc comparisons were conducted only when a significant main effect was detected (*p* < 0.05), to examine specific group differences.

An *a priori* power analysis revealed that a minimum sample size of 10 rats per group is sufficient to detect a large effect size (Cohen’s f = 0.4) with 4 groups (k = 4), a significance level of α = 0.05, and a target power of 80% (1 – β = 0.8). Notably, previous studies with psilocybin have reported significant treatment effects with fewer animals.^27^^,^^91^

Behavioral and neurobiological data were analyzed using two-way ANOVAs to examine the effects of injury (sham vs. TBI) and treatment (saline vs. psilocybin), with post-hoc Tukey tests applied where appropriate. Repeated measures ANOVAs were used for longitudinal data (e.g., body weight across months; behavioral performance across trials). For non-parametric data (i.e., nonnormal distribution or unequal variance), Kruskal-Wallis were conducted, followed by Dunn’s post-hoc tests when significant group differences were detected.

Manual VOI-based PET and MRI volumetric data was analyzed using two-way ANOVAs with group (sham/saline, sham/psilocybin, TBI/saline, and TBI/psilocybin) and brain region as factors. Tukey post-hoc tests were used where appropriate. For voxelwise analysis of group differences (sham/saline v TBI/saline and TBI/saline v TBI/psilocybin), T maps were interrogated at a *p* value of <0.01 and a cluster size of greater than 100 voxels.

Statistical significance for main effects of TBI or psilocybin treatment, as well as parametric or non-parametric post-hoc comparisons, is denoted as follows: ∗ and +, *p* < 0.05; ∗∗ and ++, *p* < 0.01; and ∗∗∗ and +++, *p* < 0.001, with ∗ indicating a significant TBI-related effect (i.e., sham/saline vs. TBI/saline or sham/psilocybin vs. TBI/psilocybin) and + indicating a significant psilocybin treatment-related effect (i.e., sham/saline vs. sham/psilocybin or TBI/saline vs. TBI/psilocybin).

## Supplemental information

Document S1. Figure S1 and Table S1

Document S2. Article plus supplemental information
