# Supplementary material for: Psilocybin restores behavior and 5-HT2A signaling while reducing microglial density after chronic traumatic brain injury in rats
Source: Cell Rep Med. 2026 Jun 12;7(7):102867. doi: 10.1016/j.xcrm.2026.102867 (PMC13400183; doi:10.1016/j.xcrm.2026.102867)
Supplement: Document S1. Figure S1 and Table S1 [file mmc1.pdf]

Cell Reports Medicine, Volume 7

Supplemental information

Psilocybin restores behavior and 5-HT_{2A} signaling while reducing microglial density after chronic traumatic brain injury in rats

Josh Allen, Bianca Jupp, Tamara L. Baker, Mohammad B. Haskali, Robert Brkljača, Zoe Plummer, Mujun Sun, Justin Brand, Brian R. Christie, Chantel T. Debert, Stuart J. McDonald, Terence J. O'Brien, Pablo M. Casillas-Espinosa, and Sandy R. Shultz

SUPPLEMENTARY INFORMATION

Table S1. Statistical information for beam training and water maze swim speed and time spent in target quadrant. No significant post-hoc group differences were observed. Graphical representations of these data are provided in Figure S1A-C.

**Behavioral statistics**	**Between-subject effects**			**Within-subject effects**			
	**TBI effect**	**Psilocybin effect**	**TBI × Psilocybin**	**Time**	**Time × TBI**	**Time × Psilocybin**	**Time × TBI × Psilocybin**
Beam training	$F_{(1, 42)}=4.053$, p=0.136	$F_{(1, 41)}=0.234$, p=0.631	$F_{(1, 41)}=0.036$, p=0.851	$F_{(5.502, 231.073)}=50.862$, **p<0.001*** **	$F_{(5.502, 231.073)}=$ 0.343, p=0.901	$F_{(5.502, 231.073)}=$ 0.915, p=0.479	$F_{(5.502, 231.073)}=$ 0.479, p=0.808
Water maze swim speed - acquisition	$F_{(1, 41)}=4.815$, **p=0.034***	$F_{(1, 41)}=0.712$, p=0.404	$F_{(1, 41)}=1.165$, p=0.287	$F_{(7.010, 287.407)}=0.586$, p=0.767	$F_{(7.010, 287.407)}=0.630$, p=0.731	$F_{(7.010, 287.407)}=1.148$, p=0.333	$F_{(7.010, 287.407)}=0.309$, p=0.950
Water maze swim speed - reversal	$F_{(1, 41)}=0.093$, p=0.762	$F_{(1, 41)}=0.405$, p=0.528	$F_{(1, 41)}=0.227$, p=0.636	$F_{(7.149, 293.109)}=5.163$, **p<0.001*** **	$F_{(7.149, 293.109)}=1.186$, p=0.310	$F_{(7.149, 293.109)}=0.283$, p=0.962	$F_{(7.149, 293.109)}=1{,}136$, p=0.340
Water maze time in target quadrant - acquisition	$F_{(1, 41)}=0.021$, p=0.884	$F_{(1, 41)}=0.117$, p=0.734	$F_{(1, 41)}=1.479$, p=0.231	$F_{(6.368, 261.092)}=1.078$, p=0.377	$F_{(6.368, 261.092)}=0.991$, p=0.434	$F_{(6.368, 261.092)}=0.635$, p=0.711	$F_{(6.368, 261.092)}=1.324$, p=0.244
Water maze time in target quadrant - reversal	$F_{(1, 41)}=3.013$, p=0.090	$F_{(1, 41)}=1.080$, p=0.305	$F_{(1, 41)}=0.290$, p=0.593	$F_{(6.957, 285.244)}=0.675$, p=0.692	$F_{(6.957, 285.244)}=0.596$, p=0.758	$F_{(6.957, 285.244)}=1.685$, p=0.113	$F_{(6.957, 285.244)}=2.302$, p=0.027

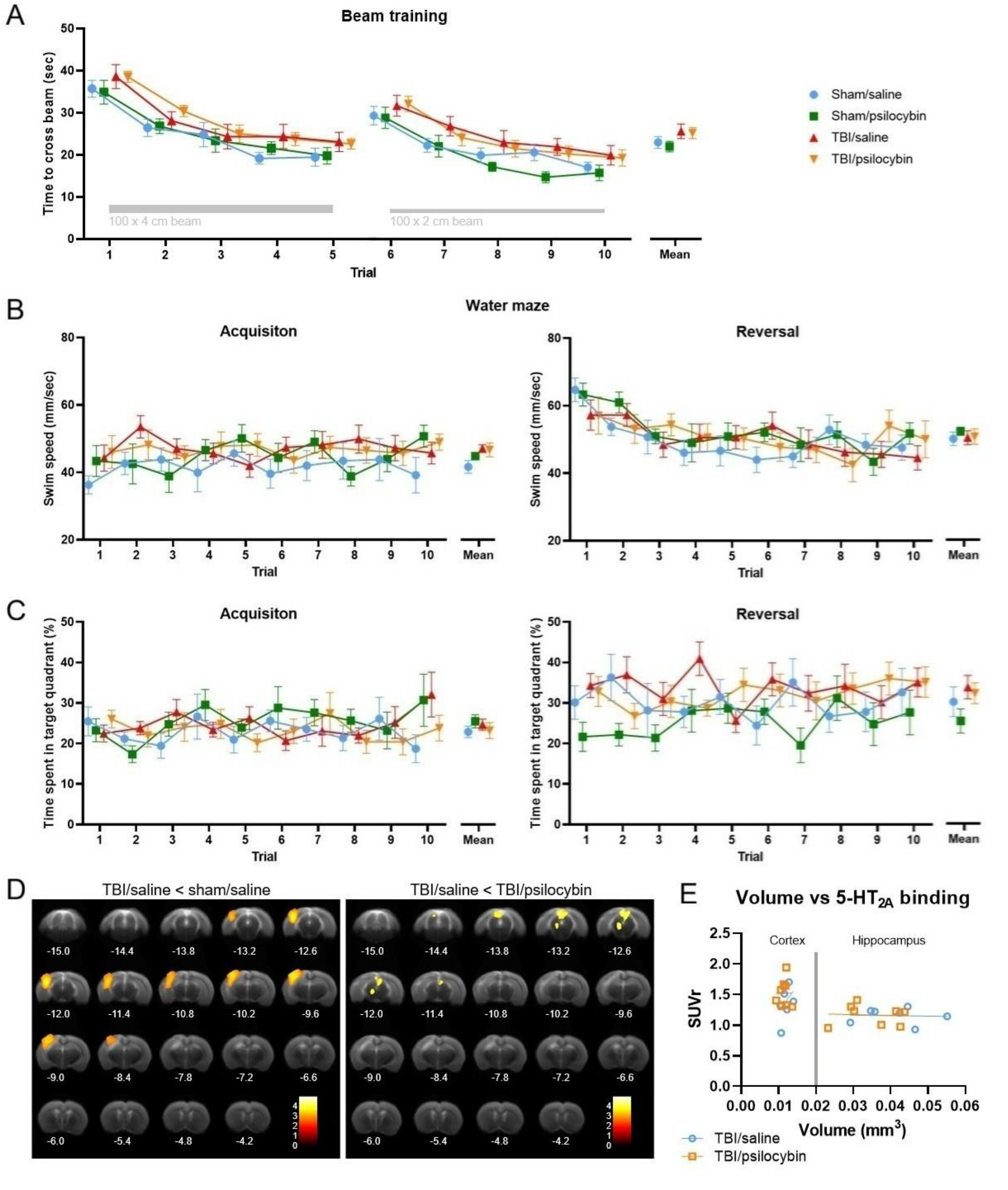

Figure S1. Beam training, water maze behavior, and volume vs 5-HT$_{2A}$ binding. A) There were no differences in beam training, which occurred the day before treatment with five trials on a 100 × 4 cm beam, followed by five on a 100 × 2 cm beam. **B)** There were no group differences in swim speed in the Acquisition or Reversal phase of the water maze. **C)** There were no group differences in percentage time spent in the target quadrant of the water maze. **D)** PET 5-HT$_{2A}$ receptor binding and MRI-derived brain volumes comparisons reveal no volumetric differences between TBI/saline and TBI/psilocybin rats, ruling out injury severity as a confound for psilocybin's effects on 5-HT$_{2A}$ binding. **E)** Exploratory voxelwise group comparisons of 5-HT$_{2A}$ binding; TBI/saline < sham/saline and TBI/saline <

TBI/psilocybin contrasts. Maps are displayed at $p<0.01$ (uncorrected) with a cluster extent threshold of 100 voxels. Colour bar represents the t-statistic. No clusters survived correction for multiple comparisons (family-wise error $p<0.05$). Data is expressed as mean±SEM. Related to Figures 2 and 3.
